# A Rapid Review on the Efficacy and Safety of Pharmacological Treatments for Chagas Disease

**DOI:** 10.3390/tropicalmed6030128

**Published:** 2021-07-12

**Authors:** Cody J Malone, Immaculate Nevis, Eduardo Fernández, Ana Sanchez

**Affiliations:** Department of Health Sciences, Faculty of Applied Health Sciences, Brock University, St. Catharines, ON L2S 3A1, Canada; cmalone@brocku.ca (C.J.M.); immaculatenevis@gmail.com (I.N.); efernandez@brocku.ca (E.F.)

**Keywords:** Chagas disease, American Trypanosomiasis, trypanocidals, rapid review

## Abstract

Chagas disease remains a neglected tropical disease, causing significant burden in the Americas and countries that receive immigrants from endemic nations. Current pharmaceutical treatments are suboptimal, not only varying drastically in efficacy, depending on the stage of disease, but also presenting significant risk of adverse events. The objective of this review is to provide a timely update on the efficacy and safety of current trypanocidals. Eligible studies published from January 2015 to December 2020 were retrieved by one reviewer from six electronic databases. Ana-lysis was done with review management software and risk of bias was assessed using tools appropriate for the type of study (i.e., experimental or observational). Thirteen studies (10 observational and three RCTs) were included in the analysis. All 13 studies tested Benznidazole (BNZ) or Nifurtimox (NFX), and two studies also tested Posaconazole (POS) or E1224 (Ravucanazole). BNZ was found to be the most efficacious trypanocidal drug compared to Nifurtimox, POS, and E1224; it also resulted in the highest percentage of adverse effects (AEs) and treatment discontinuation due to its toxicity. Adults experienced higher frequency of neurological AEs while taking BNZ or NFX compared to children. Children had a higher frequency of general AEs compared to adults while taking BNZ. Overall, BNZ is still the most efficacious, but development of new, less toxic drugs is paramount for the quality of life of patients. Studies testing combination therapies and shorter regimens are needed, as is the devising of better clinical parameters and laboratory biomarkers to evaluate treatment efficacy. Considering the variability in methodology and reporting of the studies included in the present analysis, we offer some recommendations for the improvement and replicability of clinical studies investigating pharmacological treatment of Chagas disease. These include full disclosure of methodology, standardization of outcome measures, and always collecting and reporting data on both the efficacy of trypanocidals and on safety outcomes.

## 1. Introduction

Chagas disease, scientifically known as American Trypanosomiasis, is caused by *Trypanosoma cruzi*, a hemoflagellate protozoan parasite transmitted most commonly to humans by triatomine hematophagous vectors. The infection is autochthonous to the continental Americas where favourable ecological, climatic transmission and socio-economic conditions exist. Despite great progress in its control, Chagas disease remains a significant public health problem in 21 countries in the Americas, where approximately 65–120 million people live at risk of infection, of which 6-12 million people are actually infected [1,2,3,4,5,6,7,8,9].

The current pharmaceuticals approved for the treatment of acute infection, are Benznidazole (BNZ) and Nifurtimox (NFX). Both trypanocidals were developed in the 1960–1970s [10]. These treatments are not accessible for many infected individuals, especially those already living in poverty [11]. In addition to not being widely available, even when they are, their cost prohibits fair access. For example, the total treatment cost for one patient in Colombia ranges from $46–$7981 USD per year, while in Mexico the cost is much higher, between $3000 and $14,580 USD per year [11]. These expense estimates include the cost of trypanocidals and clinical and laboratory follow-up. If treatment is not provided free of charge, this can result in unaffordability and poor adherence to the treatment regimen [11,12].

Treatment regimens with BNZ and NFX are long (often for 60 to 90 days) as well as toxic. Both trypanocidals have a significant risk of adverse effects (AEs) in about 30%–50% of those treated. Drug toxicity symptoms for BNZ and NFX include digestive intolerance, hepatitis, peripheral neuropathy, leukopenia, anorexia, weight loss, nausea, vomiting, insomnia, and dermatitis [7,13,14] These AEs can be so severe that they may result in treatment discontinuation. Compared to NFX, BNZ is typically better tolerated, more widely available, and with more support from the literature in terms of its efficacy. Thus, it is the recommended first-line treatment [6,7].

Treatment with BNZ is indicated in patients with acute Chagas disease after parasitological or serological confirmation [7]. For other forms of disease, the treatment is not straightforward. The Pan American Health Organization (PAHO) and the World Health Organization (WHO) provide a recommendations based on the quality of evidence currently available, based on their GRADE system which clinicians can use as a guideline for diagnoses and treatment [15]. As such, the PAHO/WHO have issued the following recommendations on the use trypanocidal treatment: (i) strong recommendation for their use during the acute stage, at any age; (ii) strong recommendation for children in the chronic stage; (iii) suggested use for adults in the chronic stage without specific organ damage; and (iv) not recommended use for adults with chronic infection and organ damage already established [15].

As for treatment effectiveness, this largely depends on the stage of the disease. Clinical studies have shown that the cure rate for the acute infection varies from 60%–90%, but it is only 10%–40% for the symptomatic chronic disease in adults [7,11]. Benznidazole has shown to be more effective in children aged 0-14 with chronic Chagas disease; however the majority of chronic Chagas patients are diagnosed when they are older [6,11]. In terms of treatment of patients with the chronic indeterminate form, there is no agreement as to whether trypanocidal treatment provides any benefit, as many infected individuals will never develop cardiac or GI involvement. It is hypothesized, however, that there is potential benefit in younger adults (<40–50 years of age) [7,11,12]. The current consensus indicates that trypanocidal use in individuals with established chronic chagasic cardiomyopathy is unlikely to improve clinical outcomes [7]. The debate remains as to whether or not pharmacological treatment helps prevent clinical manifestations from developing [7].

These data highlight the vital importance of studies assessing the efficacy of trypanocidals at various stages of the disease. Moreover, strong scientific evidence from up-to-date meta-analyses is needed to inform PAHO/WHO recommendations. At the time of undertaking the present review, the most recent meta-analysis on the efficacy of several trypanocidals had been conducted by Villar et al. in 2014. They found that treatment in the chronic stage of disease was more beneficial than providing no treatment, and that trypanocidal treatment has the potential to reduce heart disease progression and mortality [16].

Between 2014 and 2020, no other meta-analysis was found in the international literature. Thus, the present review aims at providing an update on the efficacy and safety of trypanocidal treatment. Unlike previous reviews and meta-analyses, the present work also expands the scope of the analysis to include children as well as all stages of disease progression.

## 2. Materials and Methods

Type of review: we conducted a rapid review, “a type of knowledge synthesis in which components of the systematic review process are simplified or omitted to produce information in a short period of time” [17,18].

Research question: we posed the following over-arching question: what is the efficacy and safety of the current and experimental trypanocidal drugs for Chagas disease compared to no treatment or placebo?

Primary outcomes:

- Any outcomes regarding efficacy of trypanocidal treatment
- Any outcomes regarding safety of trypanocidal treatment

Criteria for selecting studies:

- Types of study
  - Inclusion criteria: we included data from two study designs:
    ○ Randomized controlled trials (RCT) comparing one or more trypanocidals to an experimental group with placebo or no treatment.
    ○ Observational studies comparing those who received one trypanocidal to others who received another trypanocidal or a different dosage or no treatment, from the same or similar populations.
  - Exclusion criteria: editorials, commentaries, case-reports, conference abstracts, letters, and reviews. In addition, animal and in vitro studies, as well as studies with no comparator group(s).
- Types of participant
  Participants considered for inclusion were those infected with *T. cruzi* at any stage of the disease (acute, indeterminate, or chronic-determinate), and who had been diagnosed by positive serology and/or polymerase chain reaction (PCR).
- Types of interventions
  We considered trypanocidal treatment as any pharmaceutical intended to reduce or suppress the parasitic load, compared to a control group of no treatment, placebo, or variation in dosage.
- Adverse events
  An adverse event is defined by the U.S. Food & Drug administration (FDA) as any untoward medical occurrence during the treatment course of a drug, whether or not it is considered drug related [19]. The FDA classifies serious AEs as medical occurrences that result in death, are life-threatening, require prolonged hospitalization, or result in persistent or significant incapacitation, disruption to normal life or a congenital anomaly/birth defect [19].
- Outcome measures
  We defined two outcomes of interest
  - Infection-related: the reduction of parasitemia by PCR, sero-reversion, and/or reduction of antibody titres
  - Patient-related:
    ○ Efficacy outcomes were defined as reduction in mortality and reduction in the progression of chronic Chagas cardiomyopathy. Progression of chronic Chagas cardiomyopathy was determined as the development of cardiac abnormalities from patients that had zero abnormalities at baseline or the development of additional cardiac abnormalities for participants that had existing abnormalities at baseline
    ○ Safety outcomes were defined as any potential AE reported by a participant receiving trypanocidal treatment. The AEs were categorized as: total AEs, serious AEs, gastrointestinal, neurological, general and treatment discontinuation. AEs experienced while taking a placebo were also documented.
- Period of publication: 2015–2020.
- Language of publication: English, Spanish or Portuguese.

Search period and electronic databases: searches were run in November 2020 in the following databases: Cochrane Central Register of Controlled Trials (CENTRAL), MEDLINE (Ovid), EMBASE (Ovid), LILACS (iAHx), PubMed, SciELO. CENTRAL, MEDLINE, EMBASE and PubMed are predominately English language databases, whereas SciELO and LILACS contain more Spanish and Portuguese as well as English publications. Search terms were finalized based on results. Once the search terms were completed for one database, they were translated to other databases and explored. MeSH terms were used on applicable databases (Table S1).

Searching other sources: the top 50 Google search results for “Chagas disease treatment” were analyzed manually to determine eligibility for inclusion.

**Figure 1:**
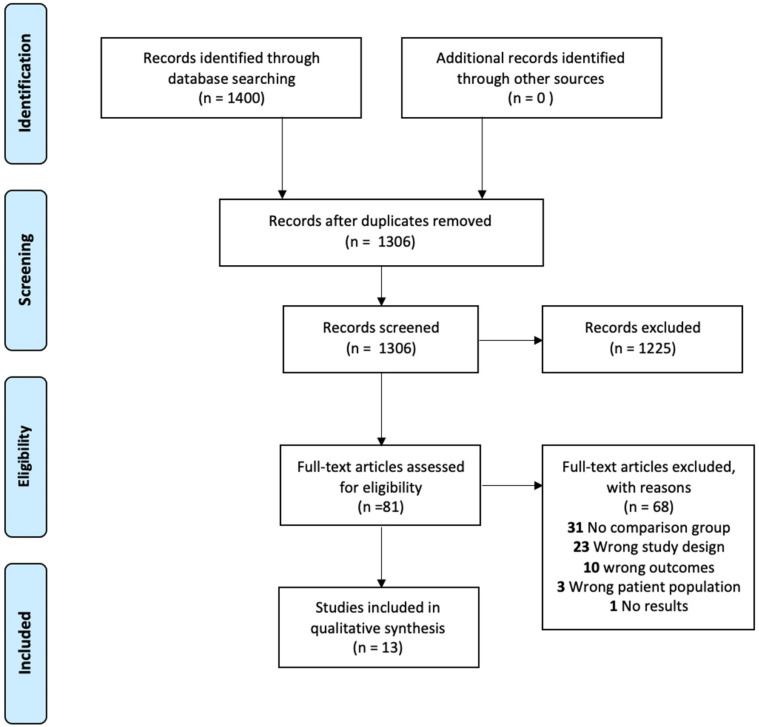
Prisma flowchart of the study selection process.

Data extraction and analysis: literature found through the searches mentioned above were imported to EndNote X9.3.3 (Clarivate analytics, 2020. London, UK.) and from there imported into a systematic review software (Covidence, v2329, 2020. Veritas Health Innovation Melbourne. Australia. Available at www.covidence.org). The identification of relevant studies was conducted on Covidence© and consisted first of screening of titles and abstracts and then the full-text of studies that were eligible. Studies were included for full-text review if the title or abstract mentioned human participants and etiological treatment of Chagas disease. The full-text was then reviewed by one reviewer (CJM) to exclude the studies that failed to meet the inclusion criteria. Data were extracted using the data extraction tool on Covidence©, which was used to collect demographic information, inclusion criteria, exclusion criteria, intervention(s), follow-up periods, conflicts of interest, and outcomes. A PRISMA flow chart of the process can be found in Figure 1.

Risk of bias assessment: the risk of bias assessment used two tools, one for non-RCTs and one for RCTs. The criteria used for the risk of bias assessment was adapted from the criteria used by Villar et al., 2014 [16] and the Cochrane ROB2 and ROBINS-I tools (Table S2).

## 3. Results

1400 articles were found through the six databases mentioned. After removing duplicates, there were 1306 unique articles (Figure 1). Of these 1306 unique articles we screened, 1225 did not meet the inclusion criteria, most of which were in vitro studies or clinical guidelines. 81 full-text studies were screened for their eligibility, 68 of which were excluded because of: no comparison group, wrong study design, wrong outcomes, wrong patient population, and/or incomplete results because the clinical trial had not finished recruiting. This left 13 studies that were eligible for inclusion; all published in English. We extracted the data and the risk of bias was assessed and is presented in Table 1**.** Detailed risk of bias assessment can be seen in Table S2.

### 3.1. Included Studies

We included three RCTs [20,21,22] and 10 observational studies [23,24,25,26,27,28,29,30,31,32]. The studies were conducted in Latin American countries or in non-endemic countries with Latin American immigrants (Table 1). Of the studies included, 10 had adult participants, two had pediatric, and one study had both pediatric and adult participants. A total of 8022 participants were included in these studies, of which 3392 received some form of trypanocidals (BNZ, NFX, POS, E1224, or a combination of two).

Of the 13 studies included, 11 reported the dosage of BNZ used [20,21,22,23,24,25,26,27,28,30,31], while the remaining two only reported “treated or untreated” based on participant response [29,32]. The most commonly used dosage of BNZ was 5 mg/kg/day orally for 60 days, with two studies using escalating doses (titration), starting at 50 mg on day one and increasing by 50 mg daily until reaching a 300 mg daily dose or in accordance with the participant’s weight **(**Table 1**)**. Three studies used NFX, with doses ranging from 8–10 mg/kg/day for 60, 84 or 90 days [23,24,32]. The median follow-up period for all studies was 4.45 years with a range of 60 days to 19.59 years.

### 3.2. Efficacy Outcomes

The trypanocidal drugs used in the included studies were BNZ, NFX, POS and E1224 (a water-soluble Ravuconazole); three studies [25,28,30] compared variations in BNZ dosing. Except for one study that initiated with acute patients, all studies participants were at some stage of indeterminate or chronic Chagas disease.

### 3.3. Infection-Related Outcomes 

Three of the 10 observational studies [23,24,25] and all the RCTs [20,21,22] reported infection-related efficacy data (Table 2). Posaconazole monotherapy and E1224 both had an antiparasitic effect during treatment as shown by parasite clearance, but this effect was not sustained after the treatment period and parasitemia returned to near placebo levels at follow-up. In the RCTs, BNZ had a statistically significant sustained efficacy compared to a placebo, E1224 or POS.

In comparison to NFX or no treatment, BNZ was also reported to be more efficacious in the observational studies [23,24], although the studies did not provide data on the comparative significance between treatment groups. Variation in BNZ treatment lengths (≤60 days and >60 days) did not show a statistically significant difference in efficacy [25].

Of the four trypanocidals used (BNZ, NFX, POS and E1224), BNZ was the most efficacious in terms of PCR conversion (clearance of parasite) and sero-reversion (quantitative decrease in anti-*T. cruzi* antibodies) in all studies that measured parasite efficacy [20,21,22,23,24].

### 3.4. Patient-Related Outcomes

Patient-related outcomes were reported in 8 of the 13 studies (two of three RCTs and six of 10 observational studies). All studies reporting patient-related outcomes had participants in the indeterminate or chronic stage of disease progression. The development and progression of ECG abnormalities were the most common patient-related measured outcome(s), with mortality being second (Table 3).

#### 3.4.1. Patient-Related Outcomes in RCTs

Neither Morillo et al., 2015 [21], nor Torrico et al., 2018 [22] found significant differences between groups in terms of development of new ECG abnormalities, prevention of cardiac clinical progression, and the primary outcome composite as seen is Table S3, used by Morillo, 2015 [20] (Table 3).

#### 3.4.2. Patient-Related Outcomes in Observational Studies

As presented in Table 3, among the observational studies, three studies [25,27,31] found no statistically significant differences between groups, while the other three studies did [26,29,32]. Table 3 also shows that Cardoso et al., 2018 [26], Fragata-Filho et al., 2016 [29], and Soverow et al., 2019 [32], observed a statistically significant difference in ECG abnormalities between treated and untreated groups [32]. Cardoso et al., 2018, and Fragata-Filho et al., 2016, also observed a statistically significant difference in mortality between treated and untreated groups [26,29].

#### 3.4.3. Safety Outcomes

The studies reported varying frequency of AEs according to the trypanocidal used. Of the 13 studies, seven presented a thorough reporting [20,21,22,23,28,29,30], two had incomplete reporting [24,25], and four did not report any AEs [26,27,31,32]. Three studies did not report AEs because it was not an outcome of interest, and one because AEs were reported in the original study conducted by Morillo et al., 2015 [20], which is also included in this review.

#### 3.4.4. Benznidazole Standard Dose (5 mg/kg/day for 60 Days)

The total frequency of treatment discontinuation observed using the standard dose of BNZ was 17.26%. With this regime, the overall frequency of AEs was 60%, comprising 11.1% serious, 27.75% gastrointestinal, 27.5% neurological, and 44% dermatological cases. Serious AEs are defined by the U.S. Food & Drug Administration as any AE that results in death, is life-threatening, requires hospitalization, causes disability or permanent damage, causes congenital abnormalities/birth defects, or requires intervention to prevent permanent damage [33]. The most common symptoms were skin rashes, dermatitis and headaches.

#### 3.4.5. Variations in Benznidazole Dosing Regimens

Three studies used a variation from the standard BNZ regime. Two of these studies thoroughly reported safety outcomes [28,30], while the third [25] reported pooled outcomes with the standard dosing regimen; and for this reason the latter was not included in the calculation of overall AEs. Treatment discontinuation in the different dosing regimens was 31%. The overall frequency of AEs was 70.5%, categorized as 9.59% serious, 11.41% gastrointestinal, 31.18% neurological, and 69.88% dermatological.

#### 3.4.6. Nifurtimox

Three studies used NFX, with one study not reporting safety outcomes [32], one reporting non-exhaustive safety results [24] and the third reporting pooled results with BNZ [23]. Treatment discontinuation was 10.7% in one study [23] and 0% in the other [24]. The incidence of AEs was only reported by one study [23], with 28.3% serious AEs, 59% gastrointestinal and 60.5% neurological. Overall, 78.7% of participants in this study reported at least one AE [23]. The four most common neurological symptoms reported were neurosensory disorders, insomnia, memory disorders, and irritability [23]. Neurological AEs were more common in adults compared to children, with 85.7% of adults experiencing at least one neurological AE whereas only 35.3% of children presented this event (*p* = 0.000) [23].

#### 3.4.7. Experimental Trypanocidals

Two studies tested experimental drugs: Morillo et al., 2017, tested Posaconazole [20] and Torrico et al., 2018, tested E1224, a water-soluble Ravuconazole [22]. Morillo et al., 2017 tested POS monotherapy as well as POS + BNZ [20]. Treatment discontinuation was 0% & 30%, respectively. For each study, the overall frequency of AEs was 62.5% and 78.6%, respectively. By category, these studies reported, respectively, 3.3% and 7.1% serious AEs; 37.5% and 35.7% gastrointestinal, 12.5% and 32.1% neurological, and 6.3% and 42.9% dermatological AEs.

The study by Torrico et al., 2018, used three dosages of E1224, low-dose, short-dose and high-dose. For each of these dosages, there were 0%, 0%, and 11% treatment discontinuation; 77%, 87%, and 73% overall frequency of AEs; 0%, 2%, and 7% serious AEs; as well as 0% neurological AEs in all three [22].

#### 3.4.8. Placebo

The three RCTs included [20,21,22] compared drug treatment against a placebo. The averages for participants receiving a placebo were: 2.3% treatment discontinuation, 47% total AEs, 1.6% serious AEs, 9.8% gastrointestinal, 4.5% neurological, and 5.7% dermatological AEs.

#### 3.4.9. Adverse Events in Adults Compared to Children

Only one study included in this review reported AEs in both children and adults [23]. Adults and children on NFX experienced a significantly higher frequency of general, gastrointestinal and neurological AEs compared to their BNZ counterparts (Table 4). In patients taking NFX, adults had a statistically higher frequency of neurological AEs than children (85.7% vs. 35.3%, *p* = 0.000) and there was a negligible difference between general and gastrointestinal AEs between age groups (*p* > 0.05). Among patients taking BNZ, children experienced a significantly higher frequency of general AEs than adults (43.5% vs. 11.7%, *p* = 0.020), but adults had a higher frequency of neurological AEs compared to children (52.9% vs. 17.4%, *p* = 0.005). In terms of gastrointestinal AEs there was no statistically significant difference between age groups (*p* > 0.05) [23].

### 3.5. Risk of Bias Assessment

The risk of bias was assessed separately for RCTs and non-RCTs, as seen in Table S2. All RCTs had a low risk of bias. Six non-RCTs had a moderate risk of bias and the final four non-RCTs had a low risk of bias.

## 4. Discussion

This review provides an update on the pharmaceutical treatment of Chagas disease. Our findings confirm that BNZ is still the most efficacious trypanocidal drug compared to other drugs (NFX, POS, and E1224) as well as to no treatment, or placebo. However, BNZ showed the highest percentage of AEs resulting in treatment discontinuation due to its toxicity.

The average frequency of treatment discontinuation for BNZ was 17.26%, which is similar to previous systematic reviews authored by Perez-Molina et al., 2009 (10%), and Villar et al., 2014 (20.5% RCTs and 10.4% observational) [16,20].

Treatment discontinuation with NFX was only reported in two studies, which were 10.7% and 0%, respectively. Other studies have reported treatment discontinuation with NFX at 20% [34], 9.9% [35], and 9.7% [36].

The lower frequency of treatment discontinuation by E1224 (0–11%) and POS (0%) may indicate a better tolerance and lower toxicity compared to BNZ. The three observational studies that examined the efficacy and safety outcomes of variations in dosing or duration found that there was no statistically significant difference in treatment efficacy or mitigation of AEs [25,28,30]. This finding concurs with the systematic review conducted by Ciapponi et al., 2020 [20].

This present study found that trypanocidal treatment in the indeterminate form of the chronic stage suggests better outcomes compared with no treatment or placebo, which was also reported in the systematic review by Villar et al., 2014 [16]. The experimental drugs E1224 and POS unfortunately did not show promise in phase 2 clinical trials as a monotherapy. Both of the drugs could potentially be used in combination therapy with BNZ because of their lower risk of AEs and treatment discontinuation [20].

The RCTs analyzed in this present study reported no statistically significant differences between treated and untreated groups in the progression of chronic Chagas cardiomyopathy or at preventing mortality. Three observational studies reported significant differences in ECG progression [26,29,32] and two observational studies reported a reduction in mortality [26,29]. It seems that trypanocidal treatment may reduce progression of abnormalities detected on ECG, and mortality, a finding that should be further explored in randomized studies with a long follow-up period.

Our findings that trypanocidal treatment during the chronic stage shows potential to reduce progression of heart disease and mortality are in agreement with those from Villar et al., 2014 [16].

Assessing the efficacy of trypanocidal treatment in the chronic stage is difficult because of the lack of a marker to define a cure [22,24,37]. The only method to define a parasitological cure at this stage is sero-reversion, which can take several years to decades to occur or may never happen. This obvious limitation makes the determination of the curative action of trypanocidals with the current serological tools available extremely difficult, if not impossible; for that reason sero-reversion should not be used as an endpoint in clinical trials [38]. The detection of parasites in the peripheral blood is a useful tool for determining if treatment was unsuccessful, but is not useful for determining a cure, as parasites may disappear in the peripheral blood and remain in the amastigote form within tissue pseudocysts [20].

The BNZ and NFX mechanisms of action are currently not fully understood, which makes it difficult to understand toxicity in order to minimize AEs [39,40]. While it is known that BNZ inhibits the parasites’ DNA and RNA, the precise mechanism remains unknown [39]. Further, it is currently suggested that Nifurtimox produces a toxic intermediate metabolite and/or a reactive oxygen species that causes DNA damage and cell death of intracellular and extracellular form of *T. cruzi*, but the precise mechanism also remains unknown [40]. Future research should be conducted to confirm the mechanisms of actions of BNZ and NFX so that AEs can potentially be mitigated. In the meantime, better management of AEs could make treatments more tolerable and reduce attrition.

### 4.1. Limitations

The present review has some methodological limitations. Firstly, the large range of follow-up periods in the included studies makes it difficult to confidently compare levels of efficacy between studies. Secondly, grey literature was not included, which may have biased our review and caused over-estimation or underestimation of our findings. Thirdly, this review used many databases, including two Latin American databases, to help mitigate selection bias, but some studies may have been missed if they were published on databases other than those searched. Fourthly, the inclusion of observational studies to answer questions about efficacy or intended effects of pharmacological treatment should be interpreted cautiously because of their inability to adequately control confounders and bias. Finally, the present study only captures literature available by December 2020. Torrico et al. [41] published the BENDITA study on April 2021 and it was therefore not included in our analysis. Future reviews should include this important study.

### 4.2. Recommendations for Future Studies

In addition to the study’s intrinsic limitations, it is worth highlighting the variability in methodology and reporting of the studies included in the present analysis. Based on these inconsistencies, we can offer the following recommendations for clinical studies investigating pharmacological treatment of Chagas disease:

(1) Disclose all methodological aspects clearly and consistently
(2) Standardize outcome measures used to enhance comparability
(3) Collect and report data on both the efficacy of trypanocidals and the safety outcomes.
(4) Report any and all AE management strategies done to mitigate toxicity and prevent treatment discontinuation.
(5) Use comparable methodology when selecting assays to assess cure or treatment failure. For instance, selecting sero-reversion alone as an indicator of parasitological cure is particularly problematic as it may take years or never occur in adult patients.
(6) When possible, studies should determine the infecting *T. cruzi* strain and make efforts to associate strain with treatment outcomes.

In addition, continued research into human polymorphisms should be encouraged to determine which polymorphisms lead to increased susceptibility to pathologies, or which may have a protective effect [42,43].

## 5. Conclusions

Benznidazole and Nifurtimox are the only currently available drugs for Chagas disease treatment and both have considerable pitfalls, including the long duration of treatment and low tolerability. Better and less toxic treatments are needed to improve adherence to treatment regimens and to improve the efficacy in non-acute stages and in individuals with overt manifestations. At present, combination therapies should continue to be explored to test their ability to lessen the duration of treatment and mitigate toxicity of BNZ or NFX. An increase in interest and funding will be required to support the discovery of new and potentially better trypanocidal drugs in the future.

Future research should aim at finding biomarkers that can be effectively used for tracking progression of clinical disease as well as markers used for assessing the curative action of trypanocidals. In addition, identifying biomarkers to predict the potential of trypanocidal treatment in order to reduce heart disease progression are urgently needed.

While different approaches to Chagas disease treatment are being developed, whether low-toxicity pharmaceuticals or a therapeutic vaccine [44]., investigating shorter treatment regimens is also warranted. In fact, a recent RCT published right after the completion of the present rapid review, the BENDITA study, by Torrico, et al., found that variations in BNZ dosing, duration, and combination therapy of BNZ with fosravucanazole were similar in efficacy regardless of the treatment duration, dose or combination therapy. The implications of this study are groundbreaking: shorter durations or lower doses of BNZ could have the same efficacy and potentially a lower risk of AEs and treatment discontinuation [41]. Future RCTs should be conducted to confirm these findings.

## Figures and Tables

**Table 1:** Characteristics of the selected studies.

Study	No. of Patients	Study Design	Country of Study (Main Nationalities of Participants)	Study Period	Treatment, Comparison and Dose
Alarcón de Noya, et al., 2017 [23]	122 (92 children & 30 adults)	Cross-sectional	Venezuela (Venezuelan)	Dec. 2007–Jan. 2010	BNZ (6 mg/kg/day) in three doses for 60 days NFX (8 mg/kg/day) for 90 days
Albareda, et al., 2018 [24]	87 (Children 5–16 yoa)	Prospective cohort	Argentina (Argentinian)	60 months (Start and end date not stated)	BNZ (5 mg/kg/day) for 60 days NFX (10 mg/kg/day) for 60 days
Antunes, et al., 2016 [25]	244 (Adults ≥ 18 yoa)	Cross-sectional	Brazil (Brazilian)	2008–2010	Had received BNZ (n = 46; 3 removed) n = 43 ^‡^NT (n = 198)
Cardoso, et al., 2018 [26]	1813 (Adults ≥ 18 yoa)	Cross-sectional (2013-2014 baseline) Cohort (2015–2016) ^†^	Brazil (Brazilian)	2013–2014 to 2015–2016 (2-year follow-up)	Had received BNZ (n = 493) NT (n = 1320)
Colantonio, et al., 2016 [27]	111 (children 6–16 yoa)	Retrospective cohort	Argentina (Argentinian)	Data used from 1991-1996 RCT (median 8.6 yr. follow-up)	BNZ (5 mg/kg/day) for 60 days Placebo for 60 days
Crespillo-Andújar, et al., 2019 [28]	471 (adults ≥ 18 yoa)	Prospective cohort	Spain Bolivian (97.5%), Paraguayan (1.5%), Salvadorian (0.5%), Honduran (0.5%)	Jan 2014–Mar 2018	Had received BNZ (5mg/kg/day) for 60 days (standard dosing scheme) BNZ (escalating dose scheme)
Fragata-Filho, et al., 2016 [29]	310 (Adults ≥ 18 yoa)	Retrospective cohort	Brazil (Brazilian)	Pre-2002 to 2013 (Average follow-up 19.59 years)	BNZ Treated (n = 263) 5 mg/kg/day for 60 days NT (n = 47)
Losada Galván, et al., 2019 [30]	62 (adults ≥ 18 yoa)	Retrospective cohort	Spain (Bolivian (97%), Honduran (3%))	July 2008-January 2017	BNZ—Full dose 5 mg/kg/day for 60 days BNZ—Escalating dose
Morillo, et al., 2015 [20]	2854 (adults 18–75 yoa)	RCT	Multiple countries Brazilian (47.6%), Argentinian (19.6%), Colombian (17.6%), Bolivian (12.5%), Salvadorian (2.7%)	2004–2011	BNZ—5 mg/kg/day for 60 days was modified in Feb. 2009 to the administration of a fixed dose of 300 mg/day and a variable duration of therapy (between 40 and 80 days) Placebo
Morillo, et al., 2017 [21]	120 (adults ≥18 to ≤ 50 yoa)	RCT	Argentina (77.5%), Chile (9.1%), Spain (8.3%), Colombia, (5%), Guatemala, (5%), Mexico (5%)	27 July 2011–24 Dec. 2013	(1) POS 400 mg b.i.d. (2) BNZ 200 mg + placebo b.i.d. (3) BNZ 200 mg b.i.d. + POS 400 mg b.i.d. (4) Placebo 10 mg b.i.d.
Schmidt, et al., 2019 [31]	1508 (adults 18–75 yoa)	Prospective sub study	Multiple Brazilian, (46.3%), Colombian, (22.3%), Argentinian, (19.2%), Bolivian (9.5%), Salvadorian (2,8%)	2004–2011	BNZ (5mg/kg/day) for 60 days or a modified regimen Placebo
Soverow, et al., 2019 [32]	89 (adults 18–60 yoa)	Prospective cohort	USA (Latin American Immigrants; nationalities not specified)	2008–2014	Dependent upon drug availability BNZ—5 mg/kg/day for 60 days (n = 18) NFX—8–10 mg/kg/day in three daily doses for 12 weeks (n = 41)
Torrico, et al., 2018 [22]	231 (adults ≥18 to ≤50 yoa)	RCT	Bolivia (Bolivian)	19 July 2011–13 June 2013	(1) High-dose E1224 (2) Short-dose E1224 (3) Low-dose E1224 (4) BNZ (5) Placebo

^‡^ Three participants terminated treatment early and were excluded; ^†^ The cross-sectional study had different outcomes of interest than the cohort study; b.i.d = twice daily; BNZ = Benznidazole, E1224 = water-soluble Ravuconazole prodrug; NFX = Nifurtimox, NT = no treatment, POS = Posaconazole, yoa: years of age.

**Table 2:** Infection-related outcomes.

**Infection-Related Efficacy (Randomized Controlled Trials)**		
**Study**		
Morillo, et al., 2015 [20]	End of treatment PCR conversion rate: BNZ 66.2% and PLA 33.5%	2-year conversion rate: BNZ 55.4% and PLA 35.3% 5-year conversion rate: BNZ 46.7% and PLA 33.1% (*p* < 0.001 for all comparisons).
Morillo, et al., 2017 [21]	RT-PCR conversion rate at 30 & 60 days: POS 93% & 90%, POS + BNZ 88.9% & 92.3%, and BNZ 89.7% & 89.3% (*p* < 0.001 for all compared to PLA)	360 day conversion was only sustained in BNZ or BNZ + POS compared with PLA and POS
Torrico, et al., 2018 [22]	End of treatment parasite clearance (PCR) (65 days): PLA 26%, LD 90%, SD 89%, HD 76% and BNZ 91% (*p* < 0.001 for all compared to PLA)	Sustained clearance at 12 months: PLA 9%, LD 8%, SD 11%, HD 29% and BNZ 82% (*p* < 0.0001)
	At 12 months follow-up analysis with conventional ELISA found no statistical difference trypanocidals and placebo	At 12 months follow-up there was a small but significant reduction in trypanolytic anti-α-gal antibodies comparing BNZ to placebo, (9% BNZ seroconverted vs. 4% of PLA) measured by CL-ELISA (*p* = 0.049)
**Infection-Related Efficacy (Observational Studies)**		
Alarcón de Noya, et al., 2017 [23]	Negative PCR conversion at follow-up (25 months): BNZ 9/10 (90%), NFX 59/112 (52.7%)	
Albareda, et al., 2018 [24]	Seroreversion ^‡^ at end of follow-up: BNZ 9/45 (20%), NFX 1/7 (14.29%)	
Antunes, et al., 2016 [25]	BNZ of ≤ 60 days and BNZ >60 days were both more efficacious than no treatment in reducing parasite load (via PCR), but no statistical differences were found between both treatments	

LD: low-dose; SD: Short-dose; HD: high-dose; PLA: Placebo; PCR: Polymerase chain reaction; RT-PCR: real time PCR; ELISA: enzyme-linked immunosorbent assay; CL-ELISA: chemiluminescent ELISA; ^‡^ As determined by sero-negativity in at least 2 of 3 tests (ELISA, Hemagglutination, and/or Immunofluorescence).

**Table 3:** Patient-related outcomes.

**Patient-Related Efficacy (RCTs)**	
Morillo et al., 2015 [20]	No significant between-group differences were observed in any component of the primary outcome No significant differences between groups with new ECG abnormalities Treatment with BNZ did not reduce cardiac clinical progression
Torrico et al., 2018 [22]	ECG outcomes were similar across treatment groups, with no clinically significant increases in QTcF during treatment.
**Patient-Related Efficacy (Observational Studies)**	
Antunes et al., 2016 [25]	No cardiac alterations were detected in the study population, regardless of group
Cardoso et al., 2018 [26]	14/493 (2.8%) of the treated group died during the 2-year follow-up; 100/1320 (7.6%) of the control group died during the 2-year follow-up (*p* ≤ 0.001) There was a reduction in well-established markers of CD severity, such as typical ECG abnormalities, high NT-proBNP levels or both. BNZ treatment reduced NT-proBNP levels.
Colantonio et al., 2016 [27]	After statistical adjustment treatment with BNZfor 60 days was not associated with less ECG abnormalities as compared to no treatment over a median follow-up of 8.6 years. The prevalence of ECGs with abnormalities was higher among children treated with BNZ compared with those not treated in all assessment periods following the baseline evaluation.
Fragata-Filho et al., 2016 [29]	20.92% of the treated patients developed ECG alterations. 3.19% of the untreated patients had worsening of ECG alterations. (*p* ≤ 0.0001) Death related to CD occurred in five participants with ECG alterations and in one with a normal ECG. (*p* = 0.001)
Schmidt et al., 2019 [31]	Those with even minimal wall motion abnormalities have poorer long-term outcomes. LV WMSI>1 was associated with a significantly increased primary outcome event rate and higher all-cause mortality (*p* ≤ 0.0001). BNZ had no significant effects on echocardiographic progression of CCC over 5.4 years.
Soverow, et al., 2019 [32]	Treated patients were less likely to have progression of their ECG disease (OR = 0.13, *p* < 0.001). Untreated patients had a higher likelihood of developing ECG abnormalities compared with their treated counterparts (56.7% vs. 11.9%, *p* ≤ 0.001).

ECG = Electrocardiogram, NT-proBNP = N-terminal of the prohormone brain natriuretic peptide, LV WMSI = Left ventricular wall motion score index, QTcF = QT interval/CubeRootRR (seconds).

**Table 4:** Comparison of adverse events among adults and children ^¥^.

Children NFX	Adults NFX	Statistical Significance
General: 75.3% [64/85] Gastrointestinal: 57.6% [46/85] Neurological: 35.3% [30/85]	General: 82.1% [23/28] Gastrointestinal: 60.7% [17/28] Neurological: 85.7% [24/28]	*p* = 0.455 *p* = 0.775 *p* = 0.000
Children BNZ	Adults BNZ	Statistical significance
General: 43.5% [20/26] Gastrointestinal: 21.7% [10/26] Neurological: 17.4% [8/28]	General: 11.7% [2/17] Gastrointestinal: 41.2% [7/17] Neurological: 52.9% [9/17]	*p* = 0.020 *p* = 0.123 *p* = 0.005
Statistical significance between NFX and BNZ General: *p* = 0.001 Gastrointestinal: *p* = 0.000 Neurological: *p* = 0.007		

^¥^ Data are from Alarcón de Noya, et al., 2017 [23].

## Data Availability

All data are contained in the article and supplementary files.

## Supplementary Materials

The following are available online at https://www.mdpi.com/article/10.3390/tropicalmed6030128/s1, Table S1—Database search strategy; Table S2—Risk of bias assessment; Table S3—Efficacy outcomes.
