# Supplementary material for: A Rapid Review on the Efficacy and Safety of Pharmacological Treatments for Chagas Disease
Source: Trop Med Infect Dis. 2021 Jul 12;6(3):128. doi: 10.3390/tropicalmed6030128 (PMC8293415; doi:10.3390/tropicalmed6030128)
Supplement: Supplementary file 1 [file tropicalmed-06-00128-s001.zip › tropicalmed-1270496-supplementary.pdf]

Table S1– Database search strategy.

	MEDLINE (OVID)	EMBASE (OVID)	LILACS	PubMed	SciELO	Cochrane CENTRAL
**Concept 1 Chagas disease**	**Headings**	**Headings**	**Headings**	**Headings**	**Keywords and phrases:**	**Headings**
	exp Chagas Disease/<br>**OR**<br>exp Trypanosomiasis/<br><br>**Keywords and phrases:**<br><br>Chagas disease.ab,ti.<br>**OR**<br>Trypanosomiasis.ab,ti.<br>**OR**<br>Chaga$.ab,ti.<br>**OR**<br>American trypanosom$.ab,ti.<br>**OR**<br>Trypanosoma cruzi.ab,ti.<br>**OR**<br>Cruzi$.ab,ti.	exp Chagas disease/<br>**OR**<br>exp trypanosomiasis/<br><br>**Keywords and phrases:**<br><br>Chagas disease.ab,ti.<br>**OR**<br>Trypanosomiasis.ab,ti.<br>**OR**<br>Chaga$.ab,ti.<br>**OR**<br>American trypanosom$.ab,ti.<br>**OR**<br>Trypanosoma cruzi.ab,ti.<br>**OR**<br>Cruzi$.ab,ti.	(mh:(c01.610.752.300.900.200))<br><br>**Keywords and phrases:**<br><br>Chagas disease<br>**OR**<br>Trypanosomiasis<br>**OR**<br>Chaga$<br>**OR**<br>American trypanosom*<br>**OR**<br>Trypanosoma cruzi*<br>**OR**<br>Cruzi*	"Chagas disease"[MeSH Terms]<br>**OR**<br>"Trypanosomiasis"[MeSH Terms]<br><br>**Keywords and phrases:**<br><br>"Chagas disease"[Title/Abstract]<br>**OR**<br>"Trypanosomiasis"<br><br>[Title/Abstract]<br>**OR**<br>"chaga*"[Title/Abstract]<br>**OR**<br>"trypanosoma cruzi"[Title/Abstract]<br>**OR**<br>"cruzi*"[Title/Abstract]	TS=(Chagas disease)<br>**OR**<br>TI=(Chagas disease)<br>**OR**<br>TS=(trypanosomiasis)<br>**OR**<br>TI=(trypanosomiasis)<br>**OR**<br>TS=(Chaga*)<br>**OR**<br>TI=(Chaga*)<br>**OR**<br>TS=(American trypanosom*)<br>**OR**<br>TI=(American trypanosom*)<br>**OR**<br>TS=(Trypanosoma cruzi)<br>**OR**<br>TI=(Trypanosoma cruzi)<br>**OR**<br>TS=(Cruzi*)<br>**OR**<br>TI=(Cruzi*)	MeSH descriptor: [Chagas Disease] explode all trees<br>**OR**<br>MeSH descriptor: [Trypanosomiasis] explode all trees<br><br>**Keyword and Phrases:**<br><br>("Chagas disease"):ti,ab,kw<br>**OR**<br>("trypanosomiasis"):ti,ab,kw<br>**OR**<br>(Chaga*):ti,ab,kw<br>**OR**<br>(American trypanosom*):ti,ab,kw<br>**OR**<br>(Trypanosoma cruzi):ti,ab,kw

**Concept 2 Trypanocids**	**Headings**	**Headings**	**Headings**	**Headings**	**Keywords and phrases:**	**Headings**
	exp Trypanocidal Agents/<br>**OR**<br>exp Nitrofurans/<br><br>**Keywords and phrases:**<br><br>Antitrypanocidal$.ab,ti.<br>**OR**<br>anti-trypanocidal$.ab,ti.**OR**<br>Trypanocid$.ab,ti.<br>**OR**<br>Antitrypanosom$.ab,ti.<br>**OR**<br>Anti-trypanosom$.ab,ti.	exp antitrypansomal agent/<br>**OR**<br>exp nitrofuran/<br>**OR**<br>exp nitrofuran derivative/<br><br>**Keywords and phrases:**<br><br>Antitrypanocidal$.ab,ti.<br>**OR**<br>Anti-trypanocidal$.ab,ti.<br>**OR**<br>Trypanocid$.ab,ti<br>**OR**<br>Antitrypanosom$.ab,ti.<br>**OR**<br>Anti-trypanosom$.ab,ti.	(mh:(d27.505.954.122.250.100.875))<br><br>**Keywords and phrases:**<br><br>antitrypanocidal*<br>**OR**<br>anti-trypanocidal*<br>**OR**<br>trypanocid*<br>**OR** antitrypanosom*<br>**OR**<br>anti-trypanosom*	"Trypanocidal agents"[MeSH Terms]<br>**OR**<br>"Nitrofurans"[MeSH Terms]<br><br>**Keywords and phrases:**<br><br>"antitrypanocidal*"[Title/Abstract]<br>**OR**<br>"trypanocid*"[Title/Abstract]<br>**OR**<br>"antitrypanosom*"[Title/Abstract]<br>**OR**<br>"anti trypanosom*"[Title/Abstract]	TS=(trypanocidal agent)<br>**OR**<br>TI=(trypanocidal agents)<br>**OR**<br>TS=(Nitrofurans)<br>**OR**<br>TI=(Nitrofurans)<br>**OR**<br>TS=(Trypanocid*)<br>**OR**<br>TI=(Trypanocid*)<br>**OR**<br>TS=(Antitrypanosom*)<br>**OR**<br>TI=(Antitrypanosom*)<br>**OR**<br>TS=(Anti-trypanosom*)<br>**OR**<br>TI=(Anti-trypanosom*)	MeSH descriptor: [Trypanocidal Agents] explode all trees<br>**OR**<br>MeSH descriptor: [Nitrofurans] explode all trees<br><br>**Keywords and Phrases:**<br><br>(Trypanocid*):ti,ab,kw<br>**OR**<br>(antitrypanosom*):ti,ab,kw<br>**OR**<br>(anti-trypanosom*):ti,ab,kw

**Final search string**	exp Chagas Disease/<br>exp Trypanosomiasis/<br>Chagas disease.ab,ti.<br>Trypanosomiasis.ab,ti.<br>American trypanosom$.ab,ti.<br>Trypanosoma cruzi.ab,ti.<br>cruzi$.ab,ti.<br>chaga$.ab,ti.<br>1 or 2 or 3 or 4 or 5 or 6 or 7 or 8<br>exp Trypanocidal Agents/<br>exp Nitrofurans/<br>antitrypanocidal$.ab,ti.<br>anti-trypanocidal$.ab,ti.<br>Trypanocid$.ab,ti.<br>antitrypanosom$.ab,ti.<br>anti-trypanosom$.ab,ti.	exp Chagas disease/<br>exp trypanosomiasis/<br>Chagas disease.ab,ti.<br>Trypanosomiasis.ab,ti.<br>Chaga$.ab,ti.<br>American trypanosom$.ab,ti.<br>Trypanosoma cruzi.ab,ti.<br>Cruzi$.ab,ti.<br>1 or 2 or 3 or 4 or 5 or 6 or 7 or 8<br>exp antitrypanosomal agent/<br>exp nitrofuran/<br>exp nitrofuran derivative/<br>antitrypanocidal$.ab,ti.<br>anti-trypanocidal$.ab,ti.<br>antitrypanosom$.ab,ti.<br>anti-trypanosom$.ab,ti.	(mh:(c01.610.752.300.900.200)) OR (chagas disease OR trypanosomiasis OR chaga* OR american trypanosom* OR trypanosoma cruzi OR cruzi*) AND (mh:(d27.505.954.122.250.100.875)) OR (antitrypanocidal* OR anti-trypanocidal* OR trypanocid* OR antitrypanosom* OR anti-trypanosom*) AND (fulltext:("1") AND db:("LILACS") AND la:("en" OR "pt" OR "es")) AND (year_cluster:[2015 TO 2020])	((((((("Chagas disease"[MeSH]) OR ("trypanosomiasis"[MeSH])) OR ((chagas disease[Title/Abstract]))) OR ((Trypanosomiasis[Title/Abstract]))) OR ((Chaga*[Title/Abstract]))) OR ((Trypanosoma cruzi[Title/Abstract]))) OR ((Cruzi*[Title/Abstract]))) AND (((((("Trypanocidal agents"[MeSH]) OR ("Nitrofurans"[MeSH])) OR ((antitrypanocidal*[Title/Abstract]))) OR ((Trypanocid*[Title/Abstract]))) OR ((Antitrypanosom*[Title/Abstract]))) OR ((anti-trypanosom*[Title/Abstract]))) Filters: Full text, English, Portuguese, Spanish, Humans, from 2015 – 2020	TS=(Chagas disease)<br>OR<br>TI=(Chagas disease)<br>OR<br>TS=(trypanosomiasis)<br>OR<br>TI=(trypanosomiasis)<br>OR TS=(Chaga*) OR<br>TI=(Chaga*) OR<br>TS=(American trypanosom*)<br>OR<br>TI=(American trypanosom*)<br>OR<br>TS=(Trypanosoma cruzi)<br>OR<br>TI=(Trypanosoma cruzi)<br>OR TS=(Cruzi*) OR<br>TI=(Cruzi*) AND<br>TS=(trypanocidal agents)<br>OR<br>TI=(trypanocidal agents) OR<br>TS=(Nitrofurans) OR<br>TI=(Nitrofurans) OR<br>TS=(Trypanocid*) OR<br>TI=(Trypanocid*) OR<br>TS=(Antitrypanosom*)<br>OR	#1 MeSH descriptor: [Chagas Disease] explode all trees<br>#2 MeSH descriptor: [Trypanosomiasis] explode all trees<br>#3 MeSH descriptor: [Trypanocidal Agents] explode all trees<br>#4 MeSH descriptor: [Nitrofurans] explode all trees<br>#5 (Trypanocid*):ti,ab,kw (Word variations have been searched)<br>#6(antitrypanosom*):ti,ab,kw (Word variations have been searched)<br>#7 (anti-trypanosom*):ti,ab,kw (Word variations have been searched)<br>#8 ("Chagas diseases"):ti,ab,kw (Word variations have been searched)<br>#9(trypanosomiasis):ti,ab,kw (Word variations have been searched)<br>#10 (Chaga*):ti,ab,kw (Word variations have been searched)

	10 or 11 or 12 or 13 or 14 or 15 or 16<br>9 and 17<br>limit 18 to (full text and humans and yr="2015 - Current")	Trypanocid$.ab, ti.<br>10 or 11 or 12 or 13 or 14 or 15 or 16 or 17<br>9 and 18<br>limit 19 to (full text and human and yr="2015 - Current")			TI=(Antitrypanosom*)<br>OR<br>TS=(Anti-trypanosom*)<br>OR<br>TI=(Anti-trypanosom*)<br>#12 OR #11 OR #10 OR<br>#9 OR #8 OR #7 OR #6<br>OR #5 OR #4 OR #3 OR #2<br>OR #1<br>#23 OR #22 OR #21 OR #20<br>OR #19 OR #18 OR #17 OR<br>#16 OR #15 OR #14<br>#24 AND #13<br>(#24 AND#13) *AND* **LANGUAGE:** (English OR Portuguese<br>OR Spanish)	#11 (American trypanosom*):ti,ab,kw (Word variations have been searched)<br>#12 (Trypanosoma cruzi):ti,ab,kw (Word variations have been searched)<br>#13 #1 OR #2 OR #8 OR #9 OR #10 OR #11 OR #11<br>#14 #3 OR #4 OR #5 OR #6 OR #7<br>#15 #13 AND #14 with Publication Year from 2015 to 2020, with Cochrane Library publication date Between Jan 2015 and Dec 2020, in Trials
**Total Results (n=1400)**	**17**	**25**	**662**	**612**	**58**	**26**

Table S2 – Risk of bias assessment*. The risk of bias was assessed depending on how many features were present (0-2, low; 3-5; moderate; 6-7, high).

Non-Randomized controlled trials

Study	Sampling/selection process described?	Eligibility criteria described?	Blinded outcome assessment?	Baseline group comparison?	Confounding management reported?	Follow-up rates described?	Adjusted results reported?	Risk of bias
Alarcón de Noya et al., 2017 [1]	Yes	Yes	No information	No	No	No	No	Moderate
Albareda et al., 2018 [2]	Yes	Yes	No	Yes	No	No	No	Moderate
Antunes et al., 2016 [3]	Yes	Yes	No information	Yes	No	Yes	No	Moderate
Cardoso et al., 2018 [4]	Yes	Yes	No information	Yes	Possible Yes	Yes	Yes	Low
Colantonio et al., 2016 [5]	Yes	Yes	Possible Yes	Yes	Yes	Yes	Yes	Low
Crespillo-Andújar et al., 2019 [6]	Yes	Yes	No	Yes	No	Yes	No	Moderate
Fragata-Filho et al., 2016 [7]	Yes	Yes	No	Yes	No	Yes	No	Moderate
Losada Galván et al., 2019 [8]	Yes	Yes	No Information	Yes	No	Yes	No	Moderate
Schmidt et al., 2019 [9]	Yes	Yes	Yes	Yes	Possible Yes	Yes	Yes	Low
Soverow et al., 2019 [10]	Yes	Yes	Yes	Yes	No	Yes	Yes	Low

Randomized controlled trials

Study	Allocation concealment described?	Randomization?	Blinding described?	Was the method of measurement appropriate?	Loss to follow-up‡ (%)	Intention-to-treat analysis?	Risk of bias
Morillo et al., 2015 [11]	No	Yes	Yes (Double)	Yes	0.5%	No	Low
Morillo et al., 2017 [12]	No	Yes	Yes (Single)	Yes	0%	Yes	Low
Torrico et al., 2018 [13]	Yes	Yes	Yes (Double)	Yes	0.8%	Yes	Low

*Adapted from Villar et al., 2014 [14].

‡Less than 10% loss to follow-up was considered acceptable.

Table S3 – Efficacy outcomes.

	No. of participants	Stage of disease progression	Treatment, comparison and dose	Efficacy outcome measure(s)	Primary efficacy outcome	Secondary efficacy outcome
**Alarcón de Noya et al., 2017 [1]**	122 (178 courses of treatment)	Acute at baseline (Dec 2007)<br><br>Indeterminate at follow-up (Jan 2011)	BNZ (6mg/kg/day) in three doses for 60 days<br><br>NFX (8mg/kg/day) for 90 days	Negative PCR conversion	122 patients treated in Dec 2007, on follow-up Jan 2010 54 remained positive. 53/112 (47.3%) for the NFX group and 1/10 (10%) from the BNZ group	
**Albareda et al., 2018 [2]**	87	"Early chronic stage"	BNZ (5mg/kg/day) for 60 days<br><br>NFX (10mg/kg/day) for 60 days	Seroreversion	10/52 (19.2%) (9 BNZ, 1 NFX) were seronegative in at least 2 of 3 tests.<br>Median seroreversion at<br>48 months	
**Antunes et al., 2016 [3]**	244	Chronic asymptomatic<br><br>Indeterminate	Received BNZ (n=46; 3 removed) n=43<br><br>(≤ 60 days, n=28; ≥ 60 days n=15)<br><br>NT (n=198)	PCR conversion and clinical cardiac alterations	<u>Qualitative PCR</u><br>The parasite load of the NT group was significantly higher (***p* = <0.05)** than that of the BNZ ≤ 60 d group but was not significantly higher than that of the BNZ > 60 d group.<br><br>BNZ ≤ 60 d vs. BNZ > 60 d **(*p* = 0.433)**<br><br><u>Quantitative PCR</u><br>The parasite load of the NT group was significantly higher than that of the BNZ groups **(*p* = <0.05)**	No alterations were detected in the study population, regardless of group

					BNZ ≤ 60 d vs. BNZ > 60 d (***p* = 0.839**)<br><br>Duration of treatment did not influence parasite load	
**Cardoso et al., 2018 [4]**	1813	"Early chronic stage"	Self-reported having received BNZ (n=493)<br><br>Most patients used the drug more than 10 years before the study interview<br><br>NT (n=1320)	Primary outcome: Reduction in mortality<br><br>Secondary outcome at baseline: ECG abnormalities	14/493 (2.8%) of the treated group died during the 2-year follow-up<br><br>100/1320 (7.6%) of the control group died during the 2-year follow-up (***p* ≤ 0.001**)	233/491 (47.3%) of participants had typical ECGs at baseline<br><br>773/1288 (60%) of participants had typical ECGs at baseline (***p* ≤ 0.001**)
**Colantonio et al., 2016 [5]**	111	Chronic stage	BNZ (5mg/kg/day) for 60 days n=48 (randomized during clinical trial) n=16 open label treatment<br><br>Placebo for 60 days (n=47)	ECG abnormalities	Of participants who didn't have ECG abnormalities at baseline, 16/86 (18.6%) developed ECG abnormalities during follow-up. 8/16 (50%) received BNZ<br><br>Adjusted hazard ratio for incident ECG abnormalities associated with BNZ treatment was 0.68 (95% CI: 0.25–1.88, ***p* = 0.46**)	After statistical adjustment treatment with BNZ for 60 days was not associated with less ECG abnormalities as compared with no treatment over a median follow-up of 8.6 years<br><br>*T. cruzi* infection among children with incident ECG abnormalities who received treatment with BNZ showed no persistent infection
**Crespillo-Andújar et al., 2019 [6]**	471	Chronic stage	Had received BNZ (5mg/kg/day) for 60 days (standard dosing scheme n=201)<br><br>BNZ - escalating dose			

			scheme (escalating dosing schedule during the first five days, from 50 mg on day one,100 mg day two, 150 mg day three, 200 mg day four to 300 mg day five, which was the maximum daily dose n=270)			
**Fragata-Filho et al., 2016 [7]**	310	Chronic stage	Treated (n=263)<br><br>Not treated (n=47)	ECG abnormalities	55/263 (20.92%) of the treated participants developed ECG alterations<br><br>25/47 (53.19%) of the untreated participants had worsening of ECG<br><br>Normal ECGs in treated (79.08%) and in untreated (46.81%) (***p* ≤ 0.0001**)	Death related to Chagas disease occurred in five participants with ECG alterations and in one with a normal ECG (*p = 0.001*) For the 171 participants with two or more IFA tests the results remained stable in untreated patients (232.72 ± 104.02 and 254.54 ± 93.41), whereas in the treated individuals, the titers decreased (144.90 ± 109.80^{+} and 70.25 ± 74.70) (***p* = 0.0001***)*
**Losada Galván et al., 2019 [8]**	62	Chronic stage	BNZ - Full dose (n=28)<br><br>BNZ - Escalating dose (n=34) (treatment was started with 50 mg per day (half a tablet) and then increased by 50 mg every day until the correct dosage according to weight was reached)			

**Morillo et al., 2015 [11]**	2854	Chronic Chagas cardiomyopathy (NYHA class I to III)	BNZ - 5mg/kg/day for 60 days was modified in February 2009 to the administration of a fixed dose of 300 mg per day and a variable duration of therapy (between 40 and 80 days) n=1431<br><br>Placebo N=1423	Primary outcome:<br><br>The first event of any of the components of the composite outcome of death, resuscitated cardiac arrest, sustained ventricular tachycardia, insertion of a pacemaker or implantable cardioverter–defibrillator, cardiac transplantation, new heart failure, stroke, or other thromboembolic event<br><br>Secondary outcome:<br><br>Negative PCR conversion<br>ECG abnormalities	The primary outcome occurred in 394/1431 (27.5%) in the BNZ group<br><br>The primary outcome occurred in 414/1423 (29.1%) in the placebo group<br><br>Unadjusted HR 0.93 (95% CI 0.81-1.07, ***p = 0.31***)<br><br>Adjusted HR 0.92 (95% CI 0.81-1.06, ***p = 0.26***)<br><br>No significant between-group differences were observed in any component of the primary outcome	Of the 1896 patients who provided a blood sample for PCR prior to randomization, results were positive in 59.5% in the BNZ group and 61.7% in the PLA group<br><br>PCR conversion rate in BNZ group was 66.2% and 33.5% in PLA group at the end of treatment, 55.4% and 35.3% at 2 years, 46.7% and 33.1% at 5 years or more (***p = <0.001*** for all comparisons)<br><br>No significant differences between groups with new ECG abnormalities<br><br>The patients' PCR status at baseline did not have a significant effect on the primary clinical outcome<br><br>BNZ significantly reduced detection of circulating parasites but did not reduce cardiac clinical progression.
**Morillo et al., 2017 [12]**	120	Chronic asymptomatic	1) POS 400 mg b.i.d.<br><br>2) BNZ 200 mg + placebo b.i.d.<br><br>3) BNZ 200 mg b.i.d. + POS 400 mg b.i.d.	<u>Primary outcome:</u> Proportion of subjects with persistent negative RT-PCR by day 180	The RT-PCR negative response rate for POS was 13.3% (95% CI: 1.2% to 25.5%) versus 10% (95% CI: 0% to 20.7%) for placebo. RT-PCR	RT-PCR conversion was only sustained in BNZ monotherapy or BNZ + POS at all time points (90, 120, 150, 180, and 360 days) compared with placebo

			4) placebo 10 mg b.i.d.	Secondary outcome:<br>Proportion of subjects with persistent negative RT-PCR by day 360	response rates of 80% (95% CI: 65.7% to 94.3%) for POS + BNZ and 86.7% (95% CI: 74.5% to 98.8%) for BNZ monotherapy<br><br>RT-PCR conversion at day 30 and 60 (end of treatment)<br><br>POS monotherapy 93% and 90%, POS + BNZ 88.9% and 92.3%, and BNZ 89.7% and 89.3%, respectively, compared with placebo (10% and 16.7%, ***p* ≤ 0.0001**).	(16.7%) and POS (23.3%) at day 360, respectively **(*p*= < *0.0001*)**
**Schmidt et al., 2019 [9]**	1508	Chronic Chagas cardiomyopathy (NYHA class I to III)	BNZ (5mg/kg/day) for 60 days or a modified regimen (n=29)<br><br>Placebo (n=30)	Primary outcome:<br>To assess the effects of BNZ on echocardiographic parameters obtained during long-term follow-up (composite of all death, resuscitated cardiac arrest, any sustained ventricular tachycardia, new or worsening symptomatic heart failure, pacemaker or implantable cardioverter-defibrillator, stroke or transient ischemic attack, systemic embolism, pulmonary embolism, and cardiac transplantation<br><br>Secondary outcome:<br>To assess which	WMSI 1≤1.5 vs. WMSI=1, HR=2.27 (1.69-3.06 95% CI) ***p = <0.0001***<br><br>WMSI >1.5 vs. WMSI=1 HR=6.42 (4.94-8.33 95% CI) ***p = <0.0001***<br><br>WMSI >1.5 had the worst prognosis<br><br>There were no significant differences between groups in the changes from base- line to the follow-up study<br><br>BNZ had no significant effects on echocardiographic progression of Chagas chronic	Subjects with no wall motion abnormalities at baseline had a better 5-year clinical prognosis<br><br>LV WMSI abnormalities were found to have a beneficial prognostic value<br><br>Those with even minimal wall motion abnormalities have poorer long-term outcomes

				echocardiographic abnormalities at baseline predicted poorer outcomes	cardiomyopathy over 5.4 years	
**Soverow et al., 2019 [10]**	89	Not reported	Dependent upon drug availability<br><br>BNZ - 5mg/kg/day for 60 days (n=18)<br><br>NFX - 8-10mg/kg/day in three daily doses for 12 weeks (n=41)	ECG disease progression	29/59 (49.15%) of the treated had an abnormal baseline ECG. 7/29 (24.13%) developed new ECG abnormalities<br><br>0/30 (0%) with normal ECG from the treated group developed an abnormal ECG<br><br>23/30 (76.66%) of the untreated had an abnormal baseline ECG. 14/23 (60.86%) developed new ECG abnormalities<br><br>3/7 (42.86%) with normal ECG from the untreated group developed an abnormal ECG	Untreated patients had a higher likelihood of developing ECG abnormalities compared with their treated counterparts (56.7% vs 11.9%, $\boldsymbol{p \leq 0.001}$)

**Torrico et al, 2018 [13]**	231	Chronic indeterminate	1) High-dose E1224 (n=45)<br><br>2) Short-dose E1224 (n=46)<br><br>3) Low-dose E1224 (n=48)<br><br>4) BNZ (n=45)<br><br>5) Placebo (n=47)	<u>Primary outcome:</u> Parasitological response to E1224 at the end of treatment, assessed by PCR<br><br><u>Secondary outcomes:</u> Parasitological response to BNZ, sustained response to 12 months, parasite clearance and load, seroreversion	Participants with parasite clearance at day 65 (end of treatment)<br><br>Placebo: 26%<br>LD E1224: 90%<br>SD E1224: 89%<br>HD E1224: 76%<br>BNZ: 91%<br><br>(***p* ≤ 0.0001** for all treatments in comparison to placebo)<br><br>After 1 week of treatment, mean qPCR repeated measurements showed a significant reduction in parasite load in all treatment groups compared with placebo. All BNZ patients cleared circulating parasite DNA after 2 weeks of treatment	Participants with sustained parasitological clearance at 12 months<br><br>Placebo: 9%<br>LD E1224: 8%<br>SD E1224: 11%<br>HD E1224: 29%<br>BNZ: 82%<br><br>The parasite load in the high-dose E1224 group remained significantly lower than in the placebo group, with no difference from the BNZ group on adjusted post-hoc comparison (*p* = 0.97, adjusted)<br><br>Change from baseline qPCR. p value comparison against PLA at 12-month follow-up: low-dose E1224 ***p* = 0.499**, short-dose E1224 ***p* = 0.744**, high-dose E1224 ***p* = 0.0015**, BNZ <*0.0001*<br><br>9% of treated (BNZ) seroconverted compared to 4% of PLA group

b.i.d: twice daily; BNZ: Benznidazole; CI: confidence interval; CL-ELISA: chemiluminescent ELISA; E1224: water-soluble ravuconazole prodrug; ECG: Electrocardiogram; ELISA: enzyme-linked immunosorbent assay; HD: high-dose; HR: Hazard ratio; LD: low-dose; LV WMSI: Left ventricular wall motion score index; NFX: Nifurtimox; NT: no treatment, PCR: Polymerase chain reaction; PLA: Placebo; POS = Posaconazole; RT-PCR: real time PCR; SD: Short-dose; WMSI = wall motion score index.

References

1. Alarcón de Noya, B.; Ruiz-Guevara, R.; Noya, O.; Castro, J.; Ossenkopp, J.; Díaz-Bello, Z.; Colmenares, C.; Suárez, J. A.; Noya-Alarcón, O.; Naranjo, L., *et al.* Long-term comparative pharmacovigilance of orally transmitted Chagas disease: First report. *Expert Rev. Anti. Infect. Ther.* 2017, 15, 319-25. 10.1080/14787210.2017.1286979.
2. Albareda, M. C.; Natale, M. A.; De Rissio, A. M.; Fernandez, M.; Serjan, A.; Alvarez, M. G.; Cooley, G.; Shen, H.; Viotti, R.; Bua, J., *et al.* Distinct treatment outcomes of antiparasitic therapy in *Trypanosoma cruzi*-Infected children is associated with early changes in cytokines, chemokines, and T-Cell phenotypes. *Front. Immunol.* 2018, 9, 10.3389/fimmu.2018.01958.
3. Antunes, A. P.; Ribeiro, A. L. P.; Sabino, E. C.; Silveira, M. F.; Oliveira, C. D. L.Botelho, A. C. d. C. Benznidazole therapy for Chagas disease in asymptomatic *Trypanosoma cruzi*-seropositive former blood donors: Evaluation of the efficacy of different treatment regimens. *Rev. Soc. Bras. Med. Trop.* 2016, 49, 713-20. 10.1590/0037-8682-0165-2016.
4. Cardoso, C. S.; Ribeiro, A. L. P.; Oliveira, C. D. L.; Oliveira, L. C.; Ferreira, A. M.; Bierrenbach, A. L.; Silva, J. L. P.; Colosimo, E. A.; Ferreira, J. E.; Lee, T. H., *et al.* Beneficial effects of Benznidazole in Chagas disease: NIH SaMi-Trop cohort study. *PLoS Negl. Trop. Dis.* 2018, 12, e0006814. 10.1371/journal.pntd.0006814.
5. Colantonio, L. D.; Prado, N.; Segura, E. L.Sosa-Estani, S. Electrocardiographic abnormalities and treatment with Benznidazole among children with chronic infection by *Trypanosoma cruzi:* A retrospective cohort study. *PLoS Negl. Trop. Dis.* 2016, 10, e0004651. 10.1371/journal.pntd.0004651.
6. Crespillo-Andújar, C.; López-Vélez, R.; Trigo, E.; Norman, F.; Díaz-Menéndez, M.; Monge-Maillo, B.; Arsuaga, M.Pérez-Molina, J. A. Comparison of the toxicity of two treatment schemes with Benznidazole for chronic Chagas disease: a prospective cohort study in two Spanish referral centres. *Clin. Microbiol. Infect.* 2019, 26, 384.e1-84.e4. 10.1016/j.cmi.2019.10.030.
7. Fragata-Filho, A. A.; França, F. F.; Fragata Cda, S.; Lourenço, A. M.; Faccini, C. C.Costa, C. A. Evaluation of parasiticide treatment with Benznidazol in the electrocardiographic, clinical, and serological evolution of Chagas disease. *PLoS Negl. Trop. Dis.* 2016, 10, e0004508. 10.1371/journal.pntd.0004508.
8. Losada Galván, I.; Madrid Pascual, O.; Herrero-Martínez, J. M.; Pérez-Ayala, A.Lizasoain Hernández, M. Does progressive introduction of Benznidazole reduce the chance of adverse events in the treatment of Chagas disease? *Am. J. Trop. Med. Hyg.* 2019, 100, 1477-81. 10.4269/ajtmh.18-0638.
9. Schmidt, A.; Dias Romano, M. M.; Marin-Neto, J. A.; Rao-Melacini, P.; Rassi, A., Jr.; Mattos, A.; Avezum, Á., Jr.; Villena, E.; Sosa-Estani, S.; Bonilla, R., *et al.* Effects of trypanocidal treatment on echocardiographic parameters in chagas cardiomyopathy and prognostic value of wall motion score index: A BENEFIT trial echocardiographic substudy. *J. Am. Soc. Echocardiogr.* 2019, 32, 286-95.e3. 10.1016/j.echo.2018.09.006.
10. Soverow, J.; Hernandez, S.; Sanchez, D.; Forsyth, C.; Flores, C. A.; Viana, G.Meymandi, S. Progression of baseline electrocardiogram abnormalities in Chagas patients undergoing antitrypanosomal treatment. *Open Forum. Infect. Dis.* 2019, 6, http://dx.doi.org/10.1093/ofid/ofz012.
11. Morillo, C. A.; Marin-Neto, J. A.; Avezum, A.; Sosa-Estani, S.; Rassi, A.; Rosas, F.; Villena, E.; Quiroz, R.; Bonilla, R.; Britto, C., *et al.* Randomized trial of benznidazole for chronic Chagas' cardiomyopathy. *N. Engl. J. Med.* 2015, 373, 1295-306. 10.1056/NEJMoa1507574.
12. Morillo, C. A.; Waskin, H.; Sosa-Estani, S.; Del Carmen Bangher, M.; Cuneo, C.; Milesi, R.; Mallagray, M.; Apt, W.; Beloscar, J.; Gascon, J., *et al.* Benznidazole and Posaconazole in eliminating parasites in asymptomatic *T. cruzi* carriers: The STOP-CHAGAS trial. *J. Am. Coll. Cardiol.* 2017, 69, 939-47. 10.1016/j.jacc.2016.12.023.
13. Torrico, F.; Gascon, J.; Ortiz, L.; Alonso-Vega, C.; Pinazo, M. J.; Schijman, A.; Almeida, I. C.; Alves, F.; Strub-Wourgaft, N.Ribeiro, I. Treatment of adult chronic indeterminate Chagas disease with Benznidazole and three E1224 dosing regimens: a proof-of-concept, randomised, placebo-controlled trial. *Lancet Infect. Dis.* 2018, 18, 419-30. 10.1016/s1473-3099(17)30538-8.
14. Villar, J. C.; Perez, J. G.; Cortes, O. L.; Riarte, A.; Pepper, M.; Marin-Neto, J. A.Guyatt, G. H. Trypanocidal drugs for chronic asymptomatic *Trypanosoma cruzi* infection. *Cochrane Database Syst. Rev.* 2014, 10.1002/14651858.CD003463.pub2.
